# Two-dimensional beam steering with tunable metasurface in infrared regime

**DOI:** 10.1515/nanoph-2021-0664

**Published:** 2022-02-02

**Authors:** Sun Il Kim, Junghyun Park, Byung Gil Jeong, Duhyun Lee, Ki-Yeon Yang, Yong-Young Park, Kyoungho Ha, Hyuck Choo

**Affiliations:** Samsung Advanced Institute of Technology, Samsung Electronics, Suwon, Republic of Korea

**Keywords:** high-k dielectric, LiDAR, metasurface, oxide semiconductor, two-dimensional scanning

## Abstract

Tunable metasurfaces can change the optical properties of incident light at will such as amplitude, phase, and polarization in a time-dependent fashion. Ultrafast switching speed and the ability for the pixel size reduction of the tunable metasurface can allow various applications such as light detection and ranging, interferometric sensors, and free space optical communications, to name a few. Although there have been successful demonstrations of the wavefront shaping using the tunable metasurface, the implementation of the two-dimensional metasurface pixel array that can be individually addressed in the optical frequency regime still remains challenging. Here, we present the *experimental demonstration* of the two-dimensional beam steering with the metasurface array by the binary phase grating in the infrared regime. The metasurface unit cell is composed of metal–dielectric–oxide structure with the indium tin oxide as an active layer, which is modulated by using the top fan-out electrodes. The metasurface array is two-dimensionally pixelated and has the phase change above 137° in the infrared regime.

## 1 Introduction

The ability to control the amplitude and phase of light is of critical importance in many applications including as a three-dimensional display, digital hologram, bio-medical imaging, and light detection and ranging (LiDAR) for autonomous driving [1]. Metasurfaces are flat layers composed of optical nano-structures and have been demonstrated to control the optical properties of light in transmission or reflection at the subwavelength scale [2]. After the substantial studies of static metasurfaces based on the size- or shape-tunings, there has been growing interest in the post-fabrication tuning of the metasurface that can modulate optical signal in time-dependent fashion, which is called the active metasurface. Various refractive index-changing materials have been exploited for active metasurfaces, including transparent conducting oxides [3], [4], [5], [6], [7], [8], III–V semiconductors [9, 10], graphene or 2D materials [11, 12], and liquid crystals [13, 14], and phase changing materials [15], [16], [17]. Especially, Mosallaei and his colleagues have substantially contributed to the fundamental theory and novel innovative devices, such as broadband continuous beam steering with time-modulated metasurfaces [18], time-varying optical vortices [19], and form-factor innovation based on the transmissive metasurface [20], to name a few.

Although the general working principle of the underlying phenomenon of the active metasurface has been elucidated, and a few studies have demonstrated the one-dimensional multi-channel operation, the implementation of the two-dimensional pixelated metasurface in the optical regime that can be individually addressed has eluded engineers so far. Unlike the one-dimensional channels, which can be easily fabricated by the electrical contacts at the ends of each antenna with semi-infinite length in one direction, the two-dimensional pixels need either the vertical interconnection access (VIA) above an active matrix or structured top fan-outs. The key challenge in the top fan-outs is the degradation of the optical property due to the presence of electrodes in the optical path. Mosallaei and his colleagues have also suggested pioneering concept for the two-dimensional gate-tunable metasurface array [21]. Inheriting this theoretical prediction, it would be of critical importance to experimentally demonstrate such concept. However, it has not been reported experimentally in the optical regime yet, in contrast to the experimental demonstration in the GHz or radio-frequency regimes [22], [23], [24]. As a different technology branch, there have been other kinds of two-dimensional beam steering method, such as mixing the wavelength tuning and the phase shifter in the integrated photonics [25, 26], one-dimensional to two-dimensional conversion through disordered media [27], addressable vertical cavity surface emitting lasers incorporated with microlens [28], and heater-based row/column addressing [29, 30].

**Figure 1:**
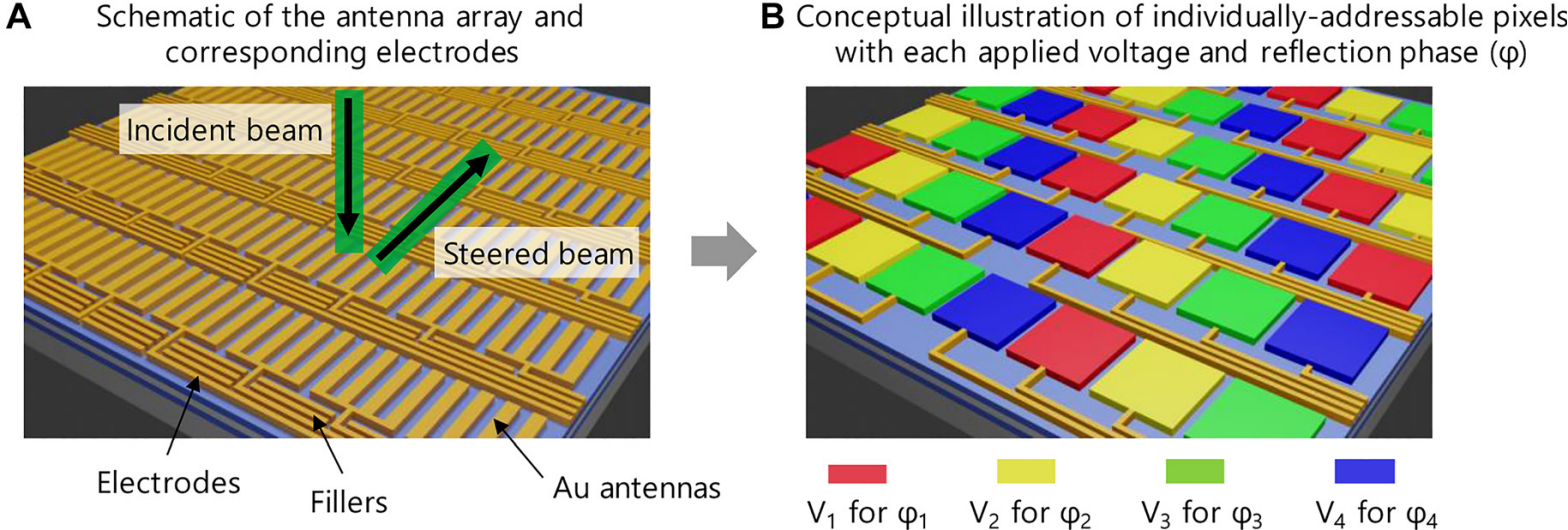
Schematic and conceptual illustration of the metasurface array for two-dimensional beam steering. (A) Schematic of the antenna array including the electrodes and fillers. The infrared incident beam illuminates the device from the top with the electrical field perpendicular to the Au antennas and produces the steered beam to the desired two-dimensional direction depending on the voltage distribution. (B) Corresponding conceptual illustration of the individually-addressable pixels with the colored patches. The colored patches do not represent the patch antenna as in Ref. [21]. The top fan-outs connected to each pixel allow the separated operation of the pixels for the given voltages.

In this article, we have presented the 10 × 10 two-dimensional metasurface pixel array with the pixel size of 5.2 × 5.2 μm^2^ that functions in the infrared regime (Figure 1A). The unit cell of our reflection-type metasurface is composed of the indium tin oxide (ITO) in the center as an active material, and the top Au antennas play two roles of the optical antenna and the electrodes. To circumvent the perturbation issue from the presence of the electrodes, we carefully design the schematic of the electrodes, which will be explained in detail below. Due to the individual electrodes defined as the top fan-outs, each pixel can be applied with each voltage *V*_i_ to represent the designed reflection phase *φ*_i_ (Figure 1B), where *i* denotes the type of voltage and phase. Here, the colored patches are used to conceptually illustrate the each antenna group, not representing the patch antennas as in Ref. [21]. Our experimental result shows the phase change above 137°, which facilitates the two-dimensional beam steering with the steering angle up to 7.3°.

## 2 Device and methodology

The active metasurface device for two-dimensional beam steering was fabricated by using the complementary metal–oxide–semiconductor (CMOS) compatible processes. First, we deposited a 10-nm-thick Ti adhesion layer on the SiO_2_/Si substrate by sputtering. Then, a Au layer with the thickness of 100 nm was adopted as a bottom mirror. The Au layer can be good mirror material because of the low surface roughness and the high reflectivity at wide infrared wavelength ranges. Then, the Au and Ti layers were patterned by a wet etching process. A 5-nm-thick ITO layer for the carrier modulation was formed by DC magnetron sputtering. The thin and uniform ITO layer was formed at the Ar/O_2_ ratio of 130/4 sccm and the deposition power of 250 W. The double layers of 1-nm-thick Al_2_O_3_ and 7-nm-thick HfO_2_ were deposited on and beneath the ITO layer by atomic layer deposition. The double layers were serially deposited in the same chamber, and H_2_O precursors were used for oxygen source. Finally, top antenna layer and fan-out electrode metal line were formed using a 50-nm-thick Au layer with a 5-nm-thick Cr adhesion layer. The patterning was achieved by e-beam lithography, followed by e-beam evaporation and a standard lift-off process.

The reflection spectrum of the fabricated active metasurface device was obtained using an optical spectrum analyzer (Yokogawa, AQ6370C) by sweeping the wavelength of the incident laser beam. The reflection phase was measured using a home-made Michelson interferometer. The two beams reflected from the active metasurface array and the reference mirror interfered and generated vertically aligned fringes, from which the phase information could be extracted. For far-field optical characterization, the metal wire bonding was carried out at all pads of the multi-channel two-dimensional active metasurface device. The laser beam (Keysight, 8164B and 81600B) was normally incident on the device, and the steered beams were measured by infrared camera (Aval, ABA-003IR-GE) according to two-dimensionally addressed applying bias. The voltages were applied to the top pixelated Au antenna, and the ITO layer was connected to the global ground.

## 3 Results and discussion

### 3.1 Simulation

We used metal–dielectric–semiconductor structure and ITO layer as an active layer for index change. When the dielectric constant of ITO layer is near zero at the interesting wavelength, strong field can be enhanced in the ITO layer. It leads the enhancement of light–matter interactions, thus increases optical modulation. The dielectric constant of ITO layer can be controlled by carrier density. When the ITO layer has the carrier density of ∼5 × 10^20^/cm^3^, the dielectric constant becomes near zero at the wavelength of 1.3 μm [1]. As we apply the electrical bias to the metal–dielectric–semiconductor structure, the charges between the ITO semiconductor layer and the Al_2_O_3_/HfO_2_ dielectric layer are accumulated under the positive bias of the top Au metal layer and are depleted under the negative bias. This change of carrier density at the interface causes the change in the dielectric constant in the ITO layer. The reflectance and phase of reflected light can be controlled by using the metasurface with these ITO layers [3].

We have designed the top Au antenna in the active metasurface device by using the finite-difference time-domain (FDTD) simulation (Lumerical™) at the resonance wavelength of 1320 nm. The period and the width of the Au antenna are 400 and 240 nm, respectively. The carrier density profiles for the applied bias were obtained by the technology-computer-aided design (TCAD) simulation (Sentaurus™).

**Figure 2:**
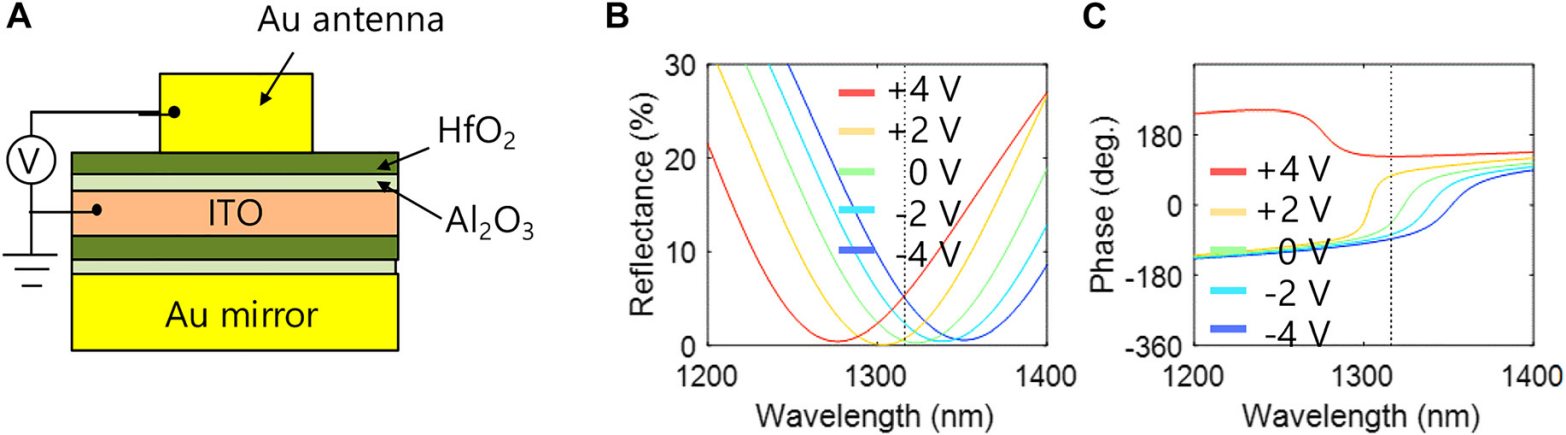
Design of the unit cell of the tunable metasurface. (A) Cross-sectional schematic diagram of the metasurface image. The thicknesses of the Au antenna, the HfO_2_ layer, the Al_2_O_3_ layer, and the ITO layer are 50 nm, 7 nm, 1 nm, and 5 nm, respectively. The incident beam is assumed to normally illuminate the structure from the top with the electric field component perpendicular to the antenna. (B) Simulated reflectivity spectrum for the Au antenna with the period of 400 nm and the width of 240 nm for the applied voltage from −4 V to +4 V with the increment step size of 2 V. (C) The corresponding phase spectrum.

Figure 2 shows the cross-sectional schematic of the metasurface and the simulated spectra of the reflectivity and reflection phase from the metasurface device with the optimal antenna design for different applied biases. The refractive index values for the HfO_2_ and Al_2_O_3_ layers were 1.91 and 1.66 from the experimental measurement, respectively. The refractive index of Au was used from the literature [31]. When a positive bias is applied on the top antenna metal, the charges are accumulated between ITO and dielectric layer, thus dielectric constant of ITO layer decreases. It leads to the blue-shift of the reflectivity and phase transition from the over-coupling to the under-coupling regimes [1, 4]. The case of depletion leads to the opposite direction due to increase in the dielectric constant. These simulation results indicate that the reflectivity and phase of the metasurface devices can be significantly reconfigured by applying voltage. We note that, the phase increases as the applied bias increases, sweeping the range above 180°. The reflectivity is around 5% at the wavelength of 1320 nm.

### 3.2 Device fabrication

**Figure 3:**
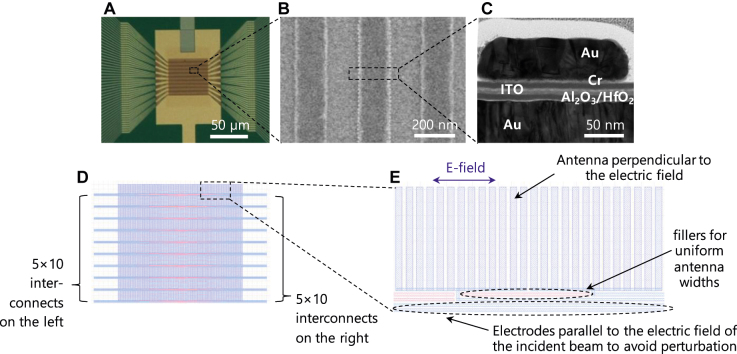
Fabrication of the 10×10 two-dimensional tunable metasurface. (A) Top microscopic image. (B) SEM image of antennas in single pixel. (C) TEM cross-sectional image in single antenna area of active metasurface array device for two-dimensional beam steering. (D) Graphic design system (GDS) layout for the ebeam patterning of both the antenna, the electrodes, and the fillers. (E) Magnified view of the GDS layout that shows the antenna perpendicular to the electric field, fillers for uniform antenna width, and the electrodes parallel to the electric field.

The active metasurface device with optimized antenna design and structure obtained from simulation was fabricated. The device has multiple pixels composed by 10 × 10 arrays that can be individually addressed and programmed for two-dimensional beam steering. We show the top view of the optical microscopic image of the active metasurface array in Figure 3A and the magnified view via the scanning electron microscopy (SEM) in Figure 3B. The cross-sectional transmission electron microscopy (TEM) images of each pixel are provided in Figure 3C.

Each pixel is connected by the top fab-out metal lines so that voltages can be individually applied as shown in Figure 1B. The top fan-out metal electrodes are carefully designed so as to have a minimum line width in order to maximize the fill factor of the antenna region in pixels as well as to minimize the perturbation to the resonance effect due to the presence of metal electrodes. The electrode has the width of 100 nm for a reliable contact, the edge-to-edge distance between neighboring electrodes is also set to be 100 nm. The antenna and the top electrode process were performed from the same metal layer (and thus the same thickness of 50 nm) and patterning process that can be monolithically obtained with a minimum line width (Figure 3D). The pixel size is 5.2 × 5.2 μm^2^, and 13 identical antennas were grouped in each pixel. In our work, we designed the electrodes to be parallel to the electric field of the incident beam in order to avoid the perturbation. The total device size is 52 × 52 μm^2^ with 10 × 10 independently-addressable pixels. The fill factor is around 92% because of the presence of the fan-out electrodes.

The cross-sectional TEM image shows that the very thin ITO and Al_2_O_3_/HfO_2_ double layers were well formed on the smooth Au bottom mirror layer, which indicates that the uniform carrier density can be modulated between the ITO and dielectric layer. As a result, the metasurface device can feature reliable electrical stability.

Figure 3E shows the magnified view of the graphic design system (GDS) layout, which includes not only the antenna array comprising the metasurface, but also the electrodes parallel to the incident electric field and the fillers that ensure the uniform antenna width. We note that these components in the layout (Figure 3E) are *substantially distinguished* from the previous theoretical research [21] in terms of three factors. First, the electrodes in the layout in [21] was perpendicular to the incident electric field (please see Figure 1A in [21]). Second, the geometrical parameters are order of magnitude different; the thickness of 5 nm and the width of 10, 20, and 40 nm. Finally, the layout in [21] did not include the fillers. It should be emphasized that these differences are the key factors that allow the successful experimental demonstration of the two-dimensional beam steering suggested by the pioneering prediction [21]. We note that experimental demonstrations of theoretically suggested previous layout often accompany with non-trivial improvement with additional requirements. For example in our work, when we tried to fabricate the electrodes with the thickness of 5 nm, we faced inevitable issues of detachment and peel-off of the electrodes. Thus, we had to increase the thickness of the electrodes to 50 nm. With the thickness of 50 nm, it is challenging to pattern the electrodes that have the width of 10 nm due to the aspect ratio. Consequently, the width is also increased. This in turn gives rise to the non-negligible perturbation of the presence of the electrodes, which was assumed to be negligible in the trailblazing theoretical suggestion in [21]. To avoid such perturbation in the real implementation, we designed the direction of the electrodes so as to be parallel to the electric field of the incident beam. In addition, despite the proximity effect correction (PEC) in the e-beam lithography, the non-uniform fill factor of the antenna and the electrodes may lead to the variation in the antenna width. We resolved such issue by adding the fillers that ensures uniform dose exposure and the uniform antenna width.

### 3.3 Optical properties and two-dimensional beam steering

**Figure 4:**
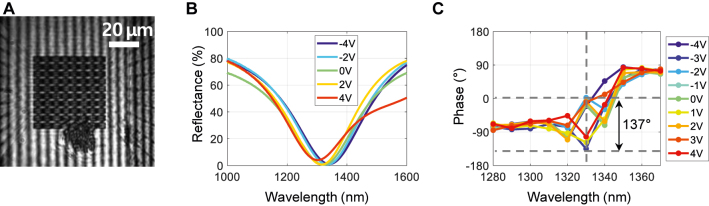
Experimental measurement of the reflection phase. (A) Captured fringe pattern in the Michelson interferometer. (B) Reflectance of the device with the applied bias from −4 V to +4 V with the step size of 2 V. (C) Reflection phase spectrum of fabricated device as a function of the wavelength ranging from 1280 to 1370 nm for the applied bias from −4 V to +4 V with the step size of 1 V, showing the phase change up to 137°.

The reflectivity and phase spectrum of the fabricated active metasurface array was measured (Figure 4). The resonance wavelength of 1332 nm and the reflectance is similar to the simulation result. It shows that there is no optical degradation or direct reflection from the top fan-out metal line. From the image captured by the infrared camera (Figure 4A), we extracted the relative translation of the fringe pattern relative to the reference region, from which the reflection phase was achieved for the applied bias. Figure 4B shows the reflectance spectrum. In Figure 4C, we observe the phase change up to 137° at wavelength of 1330 nm. The measured phase difference was slightly smaller than that obtained from simulation of ∼180°, which can be ascribed to the charge consumption at the interface of ITO and Al_2_O_3_/Hf_2_O dielectric layer. Various defects between ITO and dielectric layer could act as charge traps, and the trapped carriers may decrease the amount of the change in the dielectric constant of the ITO layer. This issue could be solved by simultaneously depositing ITO and Al_2_O_3_/Hf_2_O layers without breaking vacuum.

**Figure 5:**
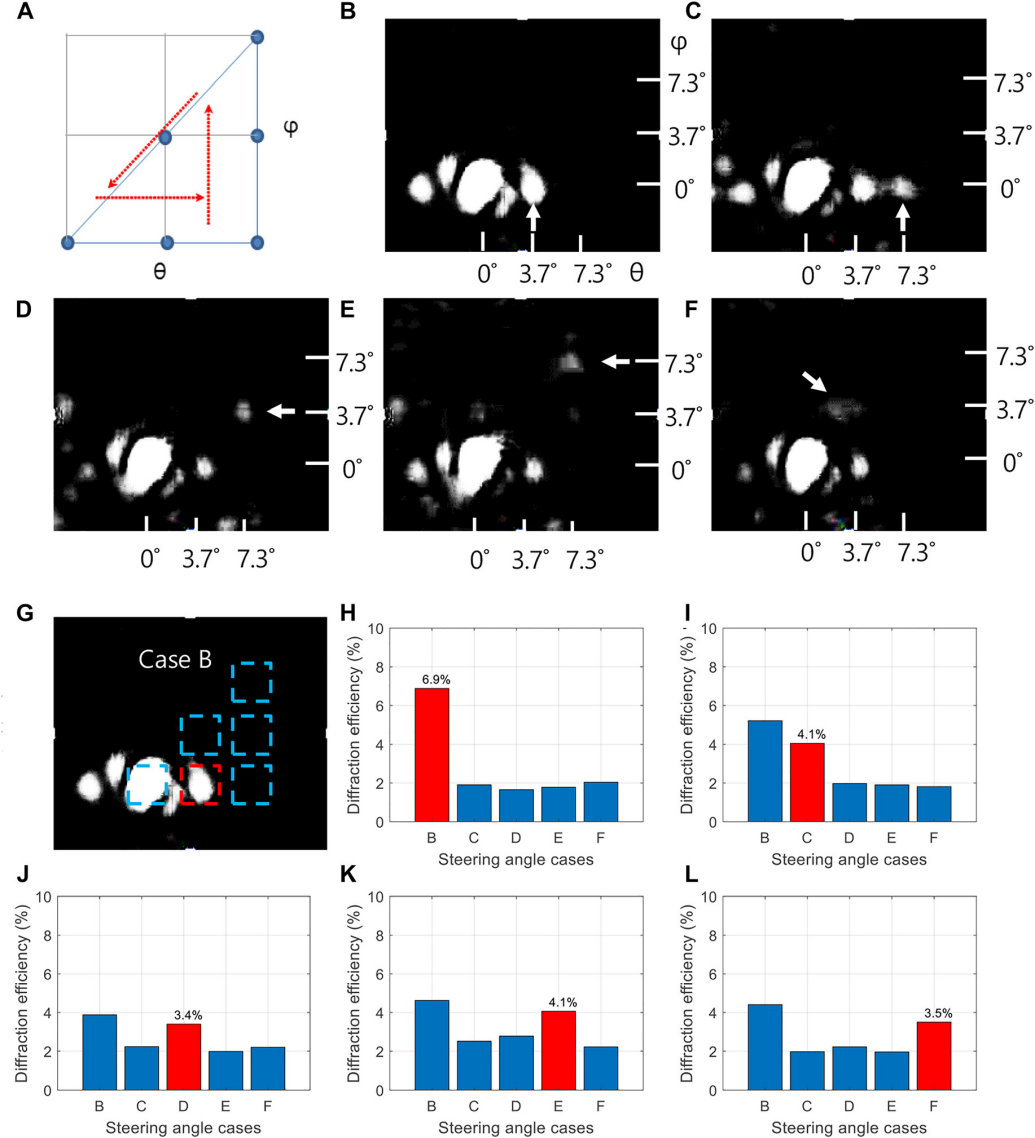
Experimental demonstration of two-dimensional beam steering. (A) The coordinate system of (*θ*, *φ*) for the beam steering spots. (B)–(F) Far-field images of light reflected from active metasurface array device, showing two-dimensional beam steering in (*θ*, *φ*) over the range of ±7.3°, i.e., the five target angles: (3.7°, 0°), (7.3°, 0°), (7.3°, 3.7°) (7.3°, 7.3°), and (3.7°, 3.7°) for (B), (C), (D), (E), and (F), respectively. (G) The steering case of (B) with the solid rectangle denoting the region of diffraction. (H)–(L) Diffraction efficiency bar plots corresponding to (B)–(F), respectively.

The two-dimensional beam steering was demonstrated by a binary phase grating (BPG) scheme with only two applied biases at the wavelength of 1330 nm. For the sake of the visualization, we depict the two-dimensional coordinate space in (*θ*, *φ*) for the beam steering angles (Figure 5A). We aim to adjust the applied voltage distribution in order to reflect the beam into desired directions in the two-dimensional space. Figure 5B–F shows the far-field images of light reflected from the active metasurface array device. The steered beam was indicated by an arrow for visibility. The bias of +4 V was applied for charge accumulation, whereas the bias of −4 V was used for depletion. These two voltages were addressed to each pixel for two-dimensional beam steering. Since the phase difference is less than 180°, several side mode beams are observed in addition to the 1st beam, showing a low side mode suppression ratio. Nonetheless, it is clearly seen that the reflected beam can move over the range of ±7.3° depending on the bias mapping. The low side mode suppression ratio seems to be the phase difference between pixels due to non-uniformity of antenna width during lift off process. There could also be optical crosstalk between pixels although the array devices are electrically modulated. This could be resolved by using array-level inverse design [6], which is beyond the scope of the current study and could remain as the future work.

Figure 5G shows the regions of interest in the far-field image. In Figure 5H–I, we show the bar plots of the diffraction efficiency corresponding to Figure 5B–F, respectively. Here, the diffraction efficiency is defined by the beam intensity in the intended region divided by the sum of all the regions denoted by rectangles in Figure 5G. It is observed that the diffraction efficiency of 6.9, 4.1, 3.4, 4.1, and 3.5% are achieved for the beam steering cases shown in Figure 5B–F. It is noteworthy that the beam at (*θ*, *φ*) = (3.7°, 0°) is always brighter than the other diffraction orders (the bar ‘B’ in Figure 5H–I). This can be ascribed to the insufficient phase difference in the experiment (137°) compared to the simulation. The absolute efficiency defined by the intensity in the intended region divided by the input beam intensity is about 0.53% compared to incident beam, and it was relatively low than previous reported paper [1]. The reason of lower efficiency and higher zeroth order beam seems to be lower phase modulation compared to the simulation of 180° and non-uniformity of phase difference between each pixel due to fabrication issues.

The diffraction order in the first (*η*_1_) and zeroth (*η*_0_) order in the BPG is given from Ref. [32] as $\eta_{1}={\left|\frac{r_{1}-r_{2}}{\pi }\right|}^{2}$ and $\eta_{0}={\left|\frac{r_{1}+r_{2}}{2}\right|}^{2}$ , respectively, where *r*_1_ and *r*_2_ denote the complex reflection coefficient from the pixel with the voltage *V*_1_ and *V*_2_, respectively. The ideal case means that two conditions are met; the magnitude of *r*_1_ and *r*_2_ should be the same (|*r*_1_| = |*r*_2_|), and the phase difference should be 180°. In such a case, *η*_0_ vanishes, and *η*_1_ becomes its maximum. The side mode suppression ratio is defined by the intensity in the desired beam normalized by the second-brightest sidelobe, which is normally the zero order. i.e., $\text{SMSR}=\frac{\eta_{1}}{\eta_{0}}$ . The reflection phase difference was measured to be △*φ* = 137°, and there could be difference in the absolute value as well. As a result the SMSR is smaller than the ideal case of infinite, and the current SMSR is around −9 to −6 dB in the experimental measurement. Such difference in the amplitude could be minimized by increasing the current fill factor of ∼92%.

## 4 Conclusions

In conclusion, we have experimentally demonstrated a 10 × 10 two-dimensional array composed of multiple metasurfaces that can modulate the phase of light in reflection in the near infrared regime (*λ* of ∼1300 nm) with the size of 5.2 × 5.2 μm^2^. Each unit cell of the metasurface consists of the ITO layer as an active material, the Au bottom mirror, and the top Au gratings. As the applied bias changes from −4 V to +4 V, the full-field simulation of the structure shows the phase change above 180°. For implementation of two-dimensional individual addressing, the top Au antennas are connected to the top fan-out electrodes, which are carefully designed so as to minimize the perturbation to the reflection property. The experimental measurement of the whole array of the metasurface shows the phase change of 137°, and we have demonstrated two-dimensional beam steering by using the BPG profile. Our work also could be extended to other materials and mechanisms for the two-dimensional pixelated metasurface. We believe these results may shed light on further research and applications such as LiDARs, free-space optical communications, and interferometric sensors.
